# Mapping the genetic basis of breast microcalcifications and their role in metastasis

**DOI:** 10.1038/s41598-018-29330-9

**Published:** 2018-07-23

**Authors:** Asif Rizwan, Santosh Kumar Paidi, Chao Zheng, Menglin Cheng, Ishan Barman, Kristine Glunde

**Affiliations:** 1 The Johns Hopkins University In Vivo Cellular and Molecular Imaging Center, Division of Cancer Imaging Research, The Russell H. Morgan Department of Radiology and Radiological Science, The Johns Hopkins University School of Medicine, Baltimore, Maryland USA; 2 Department of Mechanical Engineering, Johns Hopkins University, Baltimore, Maryland USA; 3 Department of Breast Surgery, The Second Hospital of Shandong University, Jinan, China; 4 Department of Oncology, Johns Hopkins University, Baltimore, Maryland USA; 5 The Johns Hopkins University School of Medicine, The Sidney Kimmel Comprehensive Cancer Center, Baltimore, Maryland USA

## Abstract

Breast cancer screening and early stage diagnosis is typically performed by X-ray mammography, which detects microcalcifications. Despite being one of the most reliable features of nonpalpable breast cancer, the processes by which these microcalcifications form are understudied and largely unknown. In the current work, we have investigated the genetic drivers for the formation of microcalcifications in breast cancer cell lines, and have investigated their involvement in disease progression. We have shown that stable silencing of the Osteopontin (OPN) gene decreased the formation of hydroxyapatite in MDA-MB-231 breast cancer cells in response to osteogenic cocktail. In addition, OPN silencing reduced breast cancer cell migration. Furthermore, breast cancer cells that had spontaneously metastasized to the lungs in a mouse model of breast cancer had largely elevated OPN levels, while circulating tumor cells in the same mouse model contained intermediately increased OPN levels as compared to parental cells. The observed dual roles of the OPN gene reveal the existence of a direct relationship between calcium deposition and the ability of breast cancer cells to metastasize to distant organs, mediated by common genetic factors.

## Introduction

Breast cancer is the most common malignancy in women with an incidence rate of about 120 in 100,000 women in the United States^1^. The 5 year survival rate of breast cancer patients drops from ~99% for Stage I patients, to ~27% for Stage IV disease, and thus necessitates early detection^1^. Mammography to reveal microcalcifications in the breasts is the most widely used tool in breast cancer screening and for the initial diagnosis of non-palpable breast tumors^2^. The use of microcalcifications as a reliable biomarker of breast cancer has also been questioned due to their association with both benign and malignant lesions, which leads to unnecessary biopsies^3,4^. Specifically, microcalcifications that are composed of calcium hydroxyapatite are found in both benign breast lesions and breast cancers whereas those constituted by calcium oxalate crystals are largely indicative of benign lesions. For several decades, research has mostly focused on recognizing the various morphologies that microcalcifications can have in breast tissue and their correlation with the degree of malignancy^5^. Emerging evidence from us and others suggests that higher hydroxyapatite content in mammary microcalcifications is a marker for malignant disease whereas lower hydroxyapatite and a relatively higher calcium carbonate content is characteristic of benign breast lesions^6^. Yet, such studies have provided limited information about the mechanisms governing the genesis of microcalcifications and their role in disease progression.

After having collectively been viewed as a result of cellular degeneration, a paradigm shift has recently been proposed that specific type(s) of microcalcifications are products of active cellular processes and may result from processes similar to those involved in physiological bone mineralization^7,8^. Bellahacene *et al*. reported increased expression of bone matrix proteins, which are typically involved in physiological bone mineralization, in human breast cancer cells, and speculated that they may have a role in hydroxyapatite formation^9,10^. Recently, Scimeca *et al*. showed that, under specific stimuli, epithelial cells undergoing epithelial-mesenchymal-transition (EMT) transform themselves into cells with an osteoblast-like phenotype, and are able to contribute to the production of breast microcalcifications^11^. They further demonstrated that the localization of hydroxyapatite in these cancer cells was similar to that in osteoblasts. These observations suggest that to understand the role of microcalcifications in breast cancer, it is imperative to systematically explore the genetic basis of their formation, subsequent transportation into the extracellular matrix and involvement in metastatic cancer progression.

In the current study, we seek to identify and study key genetic factors that guide the formation of microcalcifications from mammary cells, and their relationship with the migration capabilities of human breast cancer cells. To achieve this, we have examined publicly available microarray data sets for potential gene candidates in a blinded and unbiased fashion, which are differentially expressed in aggressive human breast cancer cell lines that typically develop microcalcifications *in vitro* compared to non-aggressive lines. The obtained list of candidate genes was further refined by selecting genes encoding proteins that have putative roles in tissue or cellular microcalcification. We identified the SPP1 gene encoding osteopontin (OPN) to be the most differentially expressed gene characteristic of aggressive cell lines in our list of genes. Osteopontin (OPN) is a secreted soluble glycoprotein that is present in most body fluids including milk and serum^12^. It is overexpressed in a number of different carcinomas and has previously been implicated as an enhancer of mineralization in human breast cancer samples^9^. Secreted OPN interacts with multiple cell surface receptors, including various integrins (integrin β1, integrin β3) and CD44^13^. Several studies have proposed a link between OPN and cancer^14–20^. This link, in particular to metastasis, is based on the binding of OPN to cell surface receptors such as CD44, which is critical to EMT initiation and cell-matrix adhesion in various types of primary tumors^21–23^.

Through shRNA knockdown of OPN in human MDA-MB-231 breast cancer cells, we have shown a direct involvement of the OPN gene in the formation of microcalcifications. Moreover, OPN knockdown resulted in reduced migration in *in vitro* assays, which was mediated at least in part by reduced CD44. The contribution of OPN to the migratory properties of the cancer cells was validated through *in vivo* studies by quantifying and comparing levels of OPN and CD44 expression in parental MDA-MB-231 cells orthotopically implanted in the mouse, MDA-MB-231 cells that have escaped from the primary tumor into the blood circulation, and MDA-MB-231 cells that have successfully metastasized to the lungs.

## Results

### Osteopontin expression increases with breast cancer cell aggressiveness and osteogenic cocktail treatment

**Figure 1:**
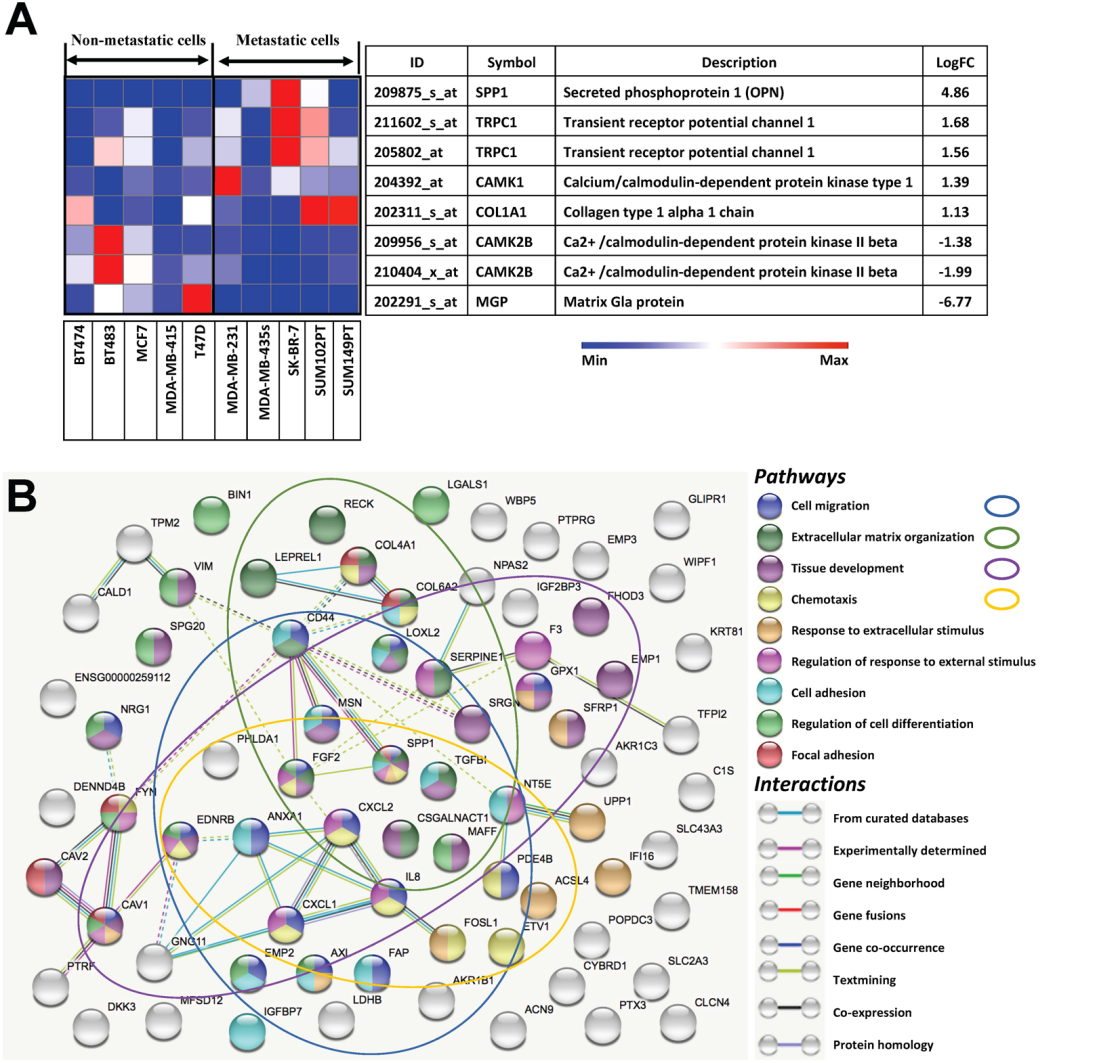
(**A**) Expression profiles of genes relevant to breast microcalcifications that are differentially expressed in metastatic and non-metastatic breast cancer cell lines. The gene expression heat map focused on genes directly linked to microcalcifications was obtained by subjecting the publicly available microarray dataset GSE16795 to the Gene-e matrix visualization and analysis platform. From the 38 cell lines included in the dataset, 5 non-metastatic and 5 metastatic cell lines with high relevance to our experimental work were identified and utilized to generate the heat map. (**B**) Protein-protein interaction network of differentially expressed genes in the selected metastatic *versus* non-metastatic cell lines. Identified biological processes and pathways that involve OPN (SPP1) are shown. Network nodes are colored by pathway membership, and pathways are sorted by increasing false discovery rate. Interactions are colored by type of interaction as listed in the legend. Solid and dotted lines represent intra-cluster and inter-cluster functional associations, respectively.

We used the GEO dataset GSE16795, which contains gene expression profiles of 39 human breast cancer cell lines, and divided it into two groups of five metastatic and five non-metastatic with high relevance to our experimental work^24^. Among the genes that are differentially expressed in metastatic *versus* non-metastatic cell lines, the genes encoding proteins with putative roles in the context of breast microcalcifications are shown in Fig. 1A in decreasing order of their log two-fold change. Figure 1A also shows the differential expression of the listed genes across the cell lines in the dataset as a heat map. Specifically, gene expression levels of OPN were found to be significantly (p-value = 0.0047) elevated (~29 fold) for the metastatic group compared to the non-metastatic group. We analyzed the protein-protein interactions of significantly differentially expressed genes in the selected metastatic *versus* non-metastatic cell lines from GSE16795 using the STRING-10.5 (http://string-db.org) analysis software and database^25^. Figure 1B visualizes a subset of the identified biological processes and pathways that involve OPN (SPP1). Network nodes are colored by pathway membership, and pathways are sorted by increasing false discovery rate. Pathways that were most significantly activated, i.e. cell migration, extracellular matrix organization, tissue development, and chemotaxis, were also circled in the same color as the corresponding nodes. Additional pathways with significant activation in metastatic cells that involve OPN were response to extracellular stimulus, regulation of response to external stimulus, cell adhesion, regulation of cell differentiation, and focal adhesion. Solid and dotted lines represent intra-cluster and inter-cluster functional associations, respectively. As seen from Fig. 1B, OPN (SPP1) directly interacts with CD44 and FGF2, which in turn interact with several other proteins, including vimentin (VIM) through CD44.

**Figure 2:**
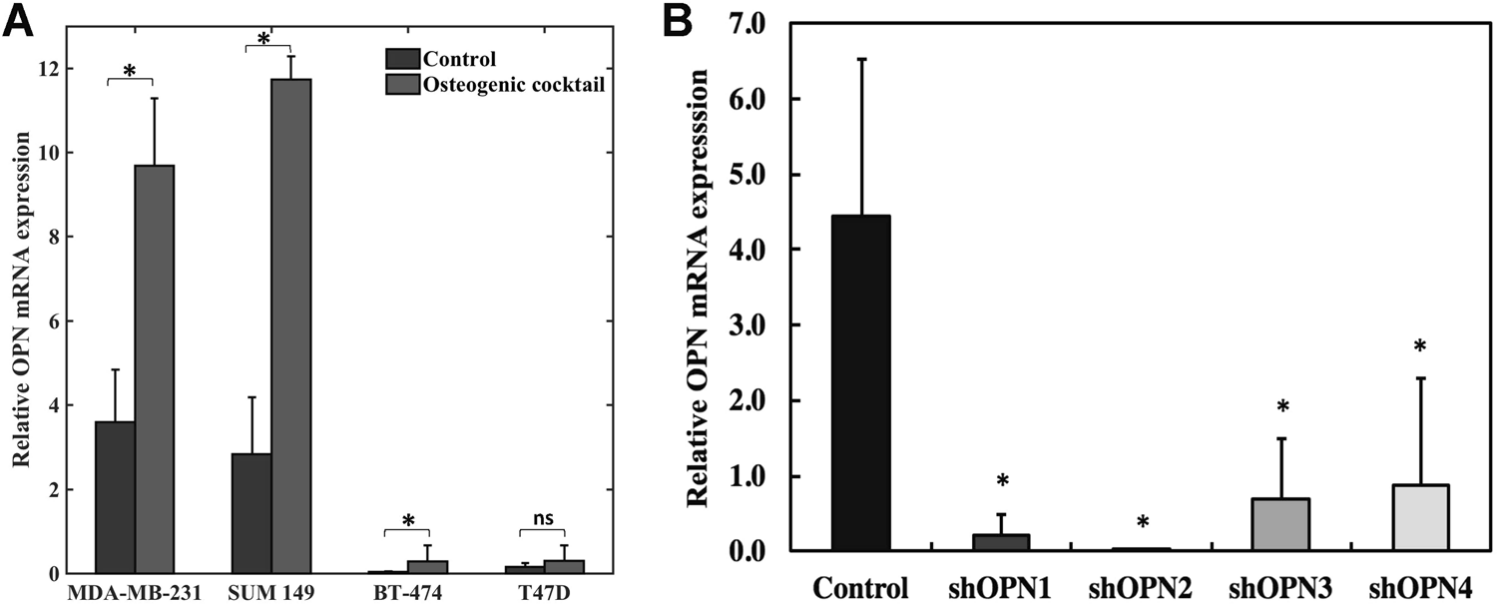
(**A**) Enhancement of OPN mRNA levels in metastatic (MDA-MB-231 and SUM 149) and non-metastatic (BT-474 and T47D) cell lines in response to addition of osteogenic cocktail. Relative expression levels of OPN mRNA in selected cell lines (analyzed using qRT-PCR) in response to osteogenic cocktail is shown. The expression levels of OPN in cells grown in control media are shown alongside for comparison. (**B**) Generation of stable clones that exhibit reduced OPN expression using shRNA silencing of the SPP1 (i.e OPN) gene in MDA-MB-231 cells. Relative expression of OPN mRNA in stably silenced clones (analyzed using qRT-PCR) in response to osteogenic cocktail is shown. Conventional Student t test threshold (p < 0.05) was considered statistically significant and is indicated by *.

As the next step, we cultured metastatic (MDA-MB-231 and SUM-149) and non-metastatic (BT-474 and T47D) human breast cancer cell lines and characterized their OPN mRNA expression levels as determined by qRT-PCR. To assess the relationship between calcification status and OPN expression level, the same cell lines were cultured in media enriched with osteogenic cocktail for induction of microcalcifications. Figure 2A shows the OPN mRNA expression results in the presence of osteogenic cocktail. The OPN mRNA expression levels of cells cultured in the absence of osteogenic cocktail are shown alongside for comparison. The OPN mRNA expression levels are significantly higher in both of the metastatic as compared to both of the non-metastatic breast cancer cell lines. It is also evident that OPN expression increases substantially with the addition of exogenous phosphates in the form of osteogenic cocktail, indicating that OPN may play a crucial role in mediating the formation of microcalcifications in breast cancer cells. The metastatic triple-negative human breast cancer cell line MDA-MB-231 was employed as the model system for further investigations in this study.

### Stable shRNA silencing of OPN inhibits the formation of cellular microcalcifications

**Figure 3:**
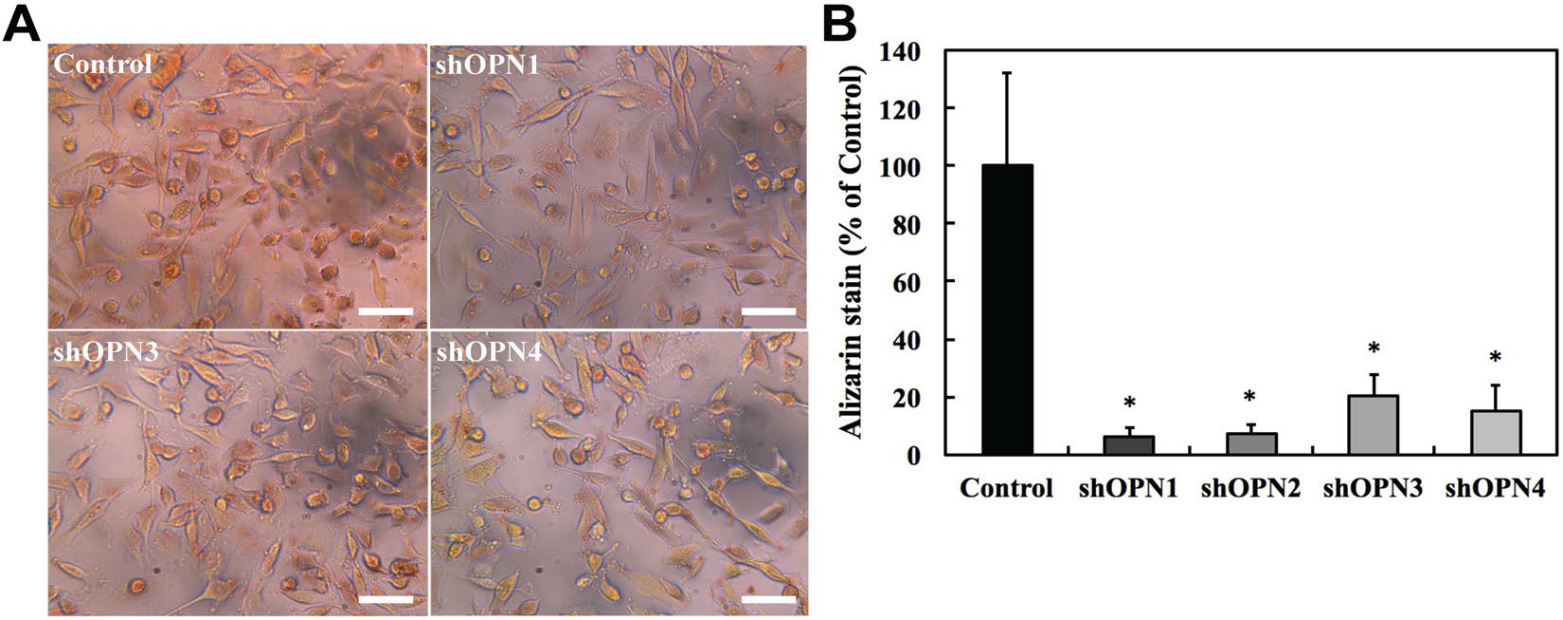
Silencing of OPN gene results in inhibition of cellular microcalcification formation in the knockdown clones. (**A**) Representative bright-field images showing alizarin red S stained cells for the four stably silenced clones and vector control. The scale bars represent 50 µm. (**B**) Bar plot showing mean and standard deviation of normalized alizarin red S stain intensity for stably silenced clones as percentage of vector control. Conventional Student t test threshold (p < 0.05) was considered statistically significant and is indicated by *.

To study the role of the OPN gene (SPP1) in the formation of microcalcifications in MDA-MB-231 cells, stable clones were generated using shRNA knockdown of the SPP1 gene in these cells. Four stable clones - shOPN1 through shOPN4 - were identified, characterized, and used for further studies. Figure 2B shows significantly reduced OPN mRNA expression levels in the stably OPN silenced lines *versus* empty-vector control cells, confirming sufficient shRNA gene knockdown. Stably silenced clones and control cells were sub-cultured in the presence of osteogenic cocktail for 7 days to induce the formation of microcalcifications. The cells were fixed and stained with alizarin red S to selectively report for the presence of microcalcifications. Figure 3A shows representative images of stably OPN silenced MDA-MB-231 clones and control cells stained with alizarin red for qualitative comparison. Three independent batches of cells were stained and the average calcification content of the cells is shown in Fig. 3B along with standard deviations. Our observations reveal that there is a consistent inhibition of the formation of cellular microcalcifications due to OPN gene silencing in the knockdown clones. The similarity in the trend of variation in level of OPN mRNA expression and cellular calcification content across the knockdown clones further strengthens our hypothesis that the OPN gene positively regulates the formation of cellular microcalcifications.

### Stable OPN silencing reduces the migration of aggressive MDA-MB-231 cells

**Figure 4:**
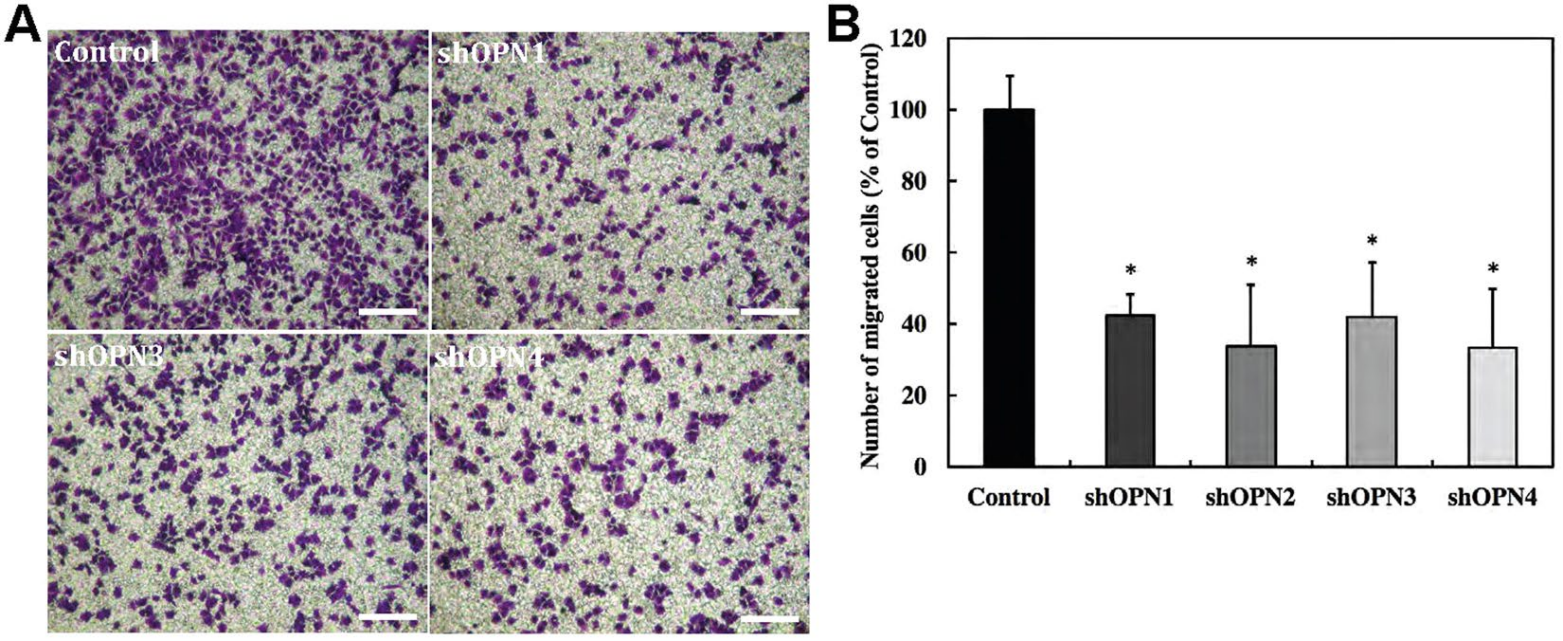
Silencing of OPN gene results in reduction of *in vitro* migration potential of the knockdown clones. (**A**) Representative bright-field images showing crystal violet stained membranes of transwell inserts for the four stably silenced clones and vector control. The scale bars represent 200 µm. (**B**) Bar plot showing mean and standard deviation of number of migrated cells for stably silenced clones as percentage of vector control. Conventional Student t test threshold (p < 0.05) was considered statistically significant and is indicated by *.

The impact of stable OPN silencing on the migration of MDA-MB-231 cells was tested using transwell migration assays. All four stably OPN silenced MDA-MB-231 lines employed in the study, shOPN1 through shOPN4, displayed reduced cell migration compared to control cells. Figure 4 shows representative migration assay images along with the quantitative comparison of migration ability for all the clones studied. The differences in migration ability were statistically significant for all clones when compared to control cells, and consistent for all the biological repeats (n = 3).

### Over-expression of OPN in circulating tumor cells (CTC) and lung metastatic cells (LMC)

**Figure 5:**
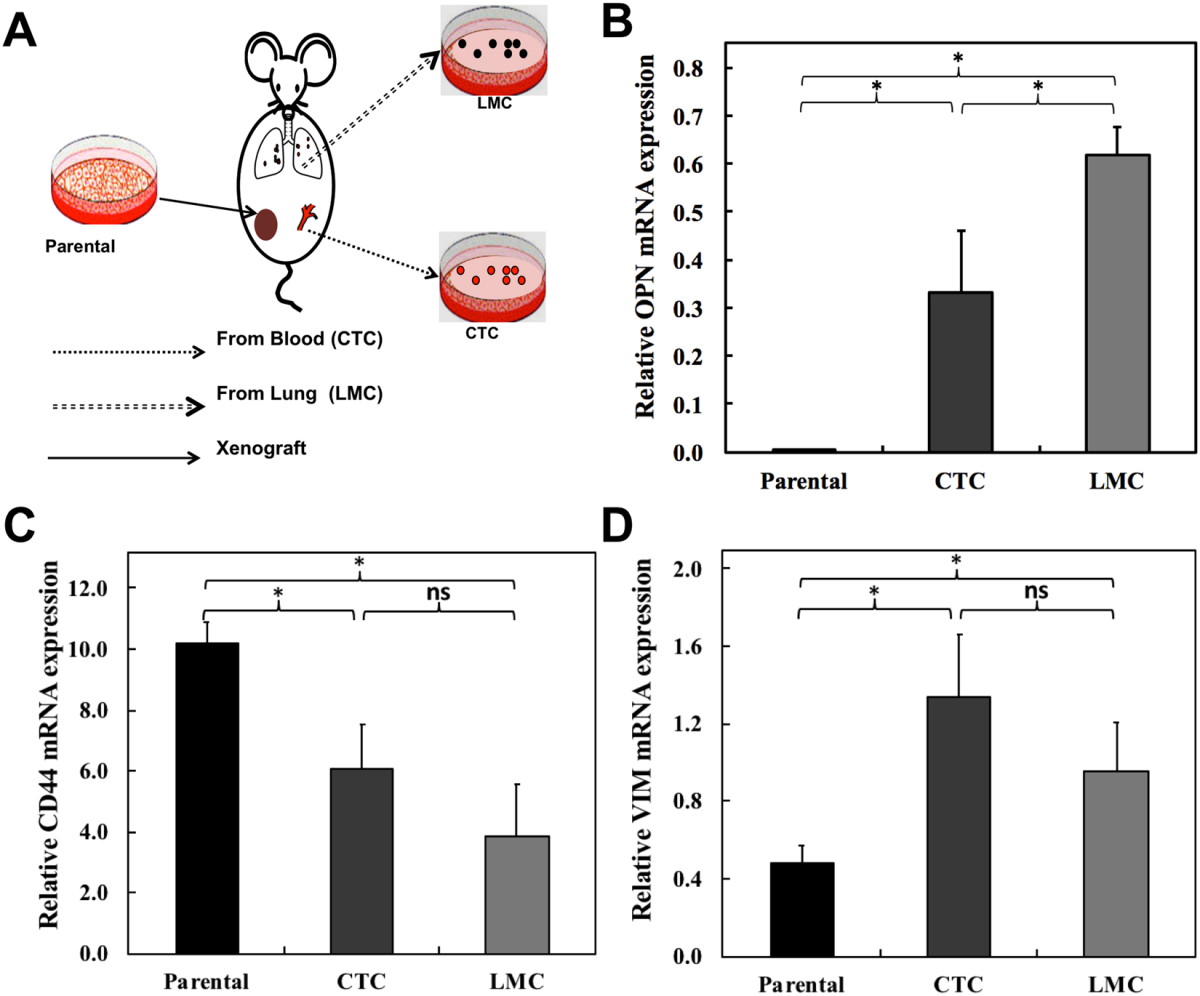
*In vivo* migration of MDA-MB-231 cells is dependent on expression of osteopontin. (**A**) Schematic of *in vivo* study conducted to isolate fluorescent tumor cells of varying metastatic potential from blood and metastatic lungs of mice carrying orthotopic MDA-MB-231 xenograft. Bar plots showing mean and standard deviation of (**B**) OPN mRNA, (**C**) CD44 mRNA, and (**D**) VIM mRNA expression for circulating tumor cells (CTCs) and lung metastatic cells (LMCs) relative to parental MDA-MB-231 cells. Conventional Student t test threshold (p < 0.05) was considered statistically significant and is indicated by *.

Motivated by the reduction of migration capabilities of the cells in the *in vitro* experiments, we performed orthotopic inoculations of MDA-MB-231 breast cancer cells and derived MDA-MB-231 tumor cells from the blood circulation (circulating tumor cells, CTC) and lungs (lung metastatic cells, LMC) at 8–12 weeks following inoculation as shown in Fig. 5A. These CTC and LMC were examined to verify the relevance of our findings *in vivo*. Figure 5B shows the mean and standard deviation of OPN mRNA expression levels comparatively for parental MDA-MB-231 cells, CTC, and LMC. The observations reveal 80-fold and 160-fold increased expression of OPN in CTC and LMC, respectively, as compared to the parental MDA-MB-231 cells. We also tested the expression levels of two of the genes identified as interacting with OPN in our protein-protein interaction analysis (Fig. 1B), which are involved in cell migration, extracellular matrix organization, and cell adhesion. The means and standard deviations of CD44 mRNA (Fig. 5C) and VIM mRNA (Fig. 5D) expression levels are shown comparatively for parental MDA-MB-231 cells, CTCs, and LMCs. We observed significant decreases in CD44 mRNA expression in CTCs and LMCs compared to parental MDA-MB-231 cells. VIM mRNA expression was significantly increased in CTCs and LMCs. Differences in CD44 and VIM mRNA levels between CTCs and LMCs were not significantly different.

## Discussion

Cellular mechanisms driving the formation of mammary microcalcifications in breast cancer cells remain unclear despite their extensive use in breast cancer screening and staging. Since the molecular biology of the formation of microcalcifications in breast cancer is poorly understood, we performed an initial *in silico* screen using an existing mRNA database for comparing highly metastatic breast cancer cell lines with non-metastatic breast cancer cell lines. In this screen, we selectively considered genes with a putative direct or indirect relationship with breast microcalcifications or bio-mineralization in general. Osteopontin, the protein encoded by the gene SPP1, which had the highest differential expression in our analysis, has previously been associated with both physiological and pathological mineralization in various organs, making it a good candidate gene for further investigation^9^. The increased expression of this gene in MDA-MB-231 cells in response to osteogenic cocktail treatment further supported the possibility of its involvement in inducing mammary mineralization. The osteogenic cocktail we used is known to induce intracellular mineralization comprising of calcium hydroxyapatite due to the increased availability of phosphate in the cell culture media with addition of β-glycerophosphate, and therefore has emerged as a popular *in vitro* model for studying mineralization^26^.

The significant suppression of intracellular mineralization as assessed by alizarin red S staining of cells cultured in osteogenic cocktail in stably OPN silenced MDA-MB-231 cells suggests that OPN is one of the principal factors directly governing cellular mineralization processes in triple-negative breast cancer cells. Studies in the past have shown that OPN is critical for both promotion and inhibition of hydroxyapatite formation in normal bone and connective tissue cells^27,28^. More recently, it has been reported that the regulation of hydroxyapatite formation depends on the phosphorylation state of OPN and its interactions with other molecules such as osteocalcin^29^. OPN also has been reported to act as hydroxyapatite nucleator when present in certain suitable conformations^29^. Based on this evidence and the results presented in this study, we reason that the formation of microcalcifications in breast cancer cells is a result of the availability of OPN in its phosphorylated state, and its dynamic interaction with several other proteins. Our observation that OPN regulates the formation of microcalcifications in breast cancer cells is in agreement with recent observations by Scimeca *et al*. that mammary microcalcifications are found in breast epithelial cells that have developed an osteoblast-like phenotype^11^. Such osteoblast-like cells developed from breast epithelial cells that were triggered by β-microglobulin to acquire mesenchymal characteristics. The osteoblast-like phenotype was sustained by bone morphogenic protein-2 (BMP-2), and these cells exhibited localized hydroxyapatite-rich cytoplasmic vesicles similar to hydroxyapatite containing intracellular vesicles found in osteoblasts^11^. In the same study, increased focal expression of OPN was observed in the proximity of hydroxyapatite, which was also a characteristic feature of lesions in biopsied tissues, showing microcalcifications along with increased mesenchymal markers such as VIM. This observation is consistent with significantly increased expression levels of the genes encoding OPN and VIM in our microarray analysis as well as in our animal model of spontaneous dissemination, in which CTCs and LMCs concurrently displayed elevated levels of OPN and VIM as compared to parental human MDA-MB-231 breast cancer cells. Together, our results support the notion that microcalcifications in aggressive breast cancer cells are driven by processes similar to those governing physiological mineralization.

Furthermore, the relationship between the microcalcification status of breast cancer cells and their metastatic capabilities remains largely unexplored. In our microarray analysis of publicly available data, we observed that OPN was significantly involved and interacted with cell migration, extracellular matrix organization, chemotaxis, and cell adhesion in metastatic breast cancer cells. Motivated by these data and the finding that microcalcifications are preferentially produced in cells displaying a mesenchymal phenotype, we assessed the effect of stable OPN knockdown on the migration capabilities of MDA-MB-231 cells. The significant inhibition of migration that we detected in all knockdown clones in *in vitro* transwell migration assays indicates that OPN expression directly affects the migration abilities of breast cancer cells. Our results are in good agreement with a recent study by Zhang *et al*., in which OPN knockdown in breast cancer cells resulted in integrin-induced inhibition of cell migration and invasion, and promoted apoptosis through induction of autophagy and inactivation of PI3K/Akt/mTOR pathway^30^. Our data also agree with recently published data showing that transient siRNA knockdown of OPN in murine mammary tumor cell lines reduced cell migration^31^.

From an orthotopic mouse xenograft model of spontaneous metastasis *in vivo*, we observed that the OPN mRNA expression level from MDA-MB-231 cells that had spontaneously metastasized to the lungs (LMC) were significantly higher than that from circulating tumor cells (CTC) from the same model, which in turn were significantly higher than that in parental cells. These progressively increasing OPN levels in MDA-MB-231 cells of the same genetic background, which have progressively traveled farther down the metastatic cascade confirms in an *in vivo* system of spontaneous metastasis that dramatically increased OPN levels are most likely required for MDA-MB-231 cells to metastasize. These observations further suggest that the OPN gene is expressed differentially in the same aggressive breast cancer cell type depending on where these cancer cells are in their metastatic journey.

Concurrently, we observed progressively decreased expression levels of CD44, which were highest in parental MDA-MB-231 cells, significantly decreased in CTCs, and the most dramatically decreased in LMCs. CD44 is a cell surface glycoprotein involved in cell communication and adhesion between adjacent cells and between cells and the extracellular matrix^32^. Reduced CD44 expression levels were previously shown to enhance breast cancer metastasis^33^, which is in good agreement with our observations. Our studies thus provide further proof for an interaction between OPN and CD44 that helps aggressive breast cancer cells to facilitate migration, spontaneous dissemination, and formation of metastatic nodules.

We also observed that the expression of VIM was significantly enhanced in CTCs and LMCs as compared to parental MDA-MB-231 cells. VIM is an intermediate filament protein that induces changes in the shape and motility of cells that are undergoing EMT^34,35^. Triple-negative breast cancers were previously shown to display elevated VIM expression levels compared to other types of breast tumors^36^. High expression levels of VIM in primary breast cancers were reported to support the formation of metastases in distant organs^37^. Taken together, our observations reinforce that an OPN-CD44-VIM interaction axis with implications in inducing EMT and reducing adhesion, helped triple-negative MDA-MB-231 cells to disseminate and form distant metastases.

Due to its active role in the regulation of several key pathways that have implications in disease progression, OPN is emerging as a novel therapeutic target^38,39^. The findings of the current study may enable accurate monitoring of response to such therapy through the evaluation of changes in microcalcification status. In addition, by exploring the relationship between OPN therapy and its impact on metastatic progression, changing microcalcification status can potentially be utilized as a marker to track metastatic development.

In conclusion, two major findings suggest the possibility of a fundamental relationship between mammary microcalcifications and metastatic capabilities of the cells in which they are formed. These findings are: (i) breast lesions rich in hydroxyapatite-based microcalcifications are associated with poor prognosis^6^, and (ii) evidence presented here that OPN is positively regulating both cellular microcalcification as well as breast cancer cell migration and metastasis, which may occur through the observed OPN-CD44-VIM axis. Future studies further exploring the causal molecular relationship between microcalcifications and metastasis in breast cancer are important for a comprehensive understanding of microcalcification etiology and their improved use as diagnostic and prognostic marker in breast cancer.

## Materials and Methods

### Identification of candidate genes responsible for breast microcalcifications

The publicly available breast cancer microarray dataset GSE 16795 was analyzed where multiple breast cancer cell lines were grown to optimal cell densities for mRNA extraction and hybridization on Affymetrix microarrays^24^. A heat map was generated using the Gene-e matrix visualization and analysis platform (http://www.broadinstitute.org). This heat map represents changes in relative content of mineralization-related gene expression levels in 5 metastatic breast cancer cell lines (MDA-MB-231, MDA-MB-435s, SK-BR-7, SUM102PT, SUM149PT) and 5 non-metastatic breast cancer cell lines (BT-474, BT-483, MCF7, MDA-MB-415, T47D).

### Protein–protein interaction network

The prominent genes overexpressed in the selected metastatic cell lines in the Gene-e analysis were employed to visualize protein-protein interactions using the STRING-10.5 (http://string-db.org) computational tool and database with a high confidence interval of 0.7^25^. The STRING network, composed of the functional protein associations is based on genomic context, high-throughput experiments, co-expression, and scientific reports. Functional enrichments in the network were identified using the STRING tool, and a subset of the identified biological processes and pathways that involve OPN (SPP1) were selected for visualization. The nodes in the network are colored according to their membership in each of the identified pathways and the pathways are sorted in the legend by increasing false discovery rates.

### Cell culture

The human breast cancer cell lines MDA-MB-231, SUM 149, BT-474 and T47D were obtained from the American Type Culture Collection (ATCC, MD)^40^. All cell lines were cultured in RPMI 1640 (Sigma-Aldrich) supplemented with 10% fetal bovine serum, 100 U/ml penicillin, 100 µg/ml streptomycin and 2 µg/ml fungizone antimycotic (Life Technology, Grand Island, NY, USA) and maintained in a humidified incubator in 5% CO_2_ at 37 °C. In a subset of the studies, the cell culture media was supplemented with an osteogenic cocktail containing 10 mM β-glycerophosphate (Sigma-Aldrich) and 50 mg/ml^-1^ ascorbic acid (Sigma-Aldrich) for promoting the formation of microcalcifications.

### Generation of stably OPN silenced breast cancer cell lines

MDA-MB-231 cells were transfected with lentiviral particles expressing shRNA against human OPN (sc-36129-V, Santa Cruz Biotechnology, Dallas, Texas) to specifically knockdown the expression of the human OPN gene. These OPN shRNA lentiviral particles were purchased as a pool of concentrated, transduction-ready viral particles containing 3 target-specific constructs that encode 19–25 nt (plus hairpin) shRNA designed to knock down OPN gene expression. Stably transduced clones were developed, along with a vector control cell line expressing a control shRNA lentiviral particle (sc-108080, Santa Cruz Biotechnology). qRT-PCR to measure OPN mRNA and immunoblotting of OPN confirmed successful transduction. For stably expressing lines, transfected cells were passaged and maintained in media containing Puromycin dihydrochloride (sc-108071, Santa Cruz Biotechnology). Cells were kept under selection for 7–10 days. Then, the cells from the selection step were plated at a density of 10 cells per ml in a 96-well tissue culture plate by adding 100 µl per well (i.e., 1 cell per well). Selected single-colony wells in the 96-well tissue culture plate were expanded to high confluence and transferred to a 24-well tissue culture plate. Once the colonies in 24-well tissue culture plates were expanded to high confluence, they were passaged to a 6-well tissue culture plate. Clonal cell lines were assessed by qRT-PCR to select lines with significantly decreased levels of OPN gene expression.

### qRT-PCR

Three cell lines from each group (MDA-MB-231, CTC, LM) were analyzed for gene expression with two technical repeats and two biological repeats each. For RNA purification, cells were grown for 48 hours in exponential growth phase and mRNA was isolated and purified using the RNeasy total RNA isolation kit (Qiagen, Germantown, MD) according to the manufacturer’s protocol. mRNA was reverse transcribed into cDNA using qScript™ cDNA SuperMix (Quanta Bioscience, Gaithersburg, MD). Quantitative real-time PCR (q-RT-PCR) was performed using IQ SYBR Green Supermix and gene-specific primers in the iCycler RT-PCR detection system (Bio-Rad, Hercules, CA) with 2 µl of diluted cDNA samples (1:10) used as a template using the following primers. The sequences for forward and reverse primers for quantifying SPP1 mRNA, which expresses the OPN protein, were 5′-CGAGGTGATAGTGTGGTTTATGG-3′ and 5′-GCACCATTCAACTCCTCGCTTTC-3′, respectively. The sequences for forward and reverse primers for quantifying CD44 mRNA were 5′-CGGACACCATGGACAAGTTT-3′ and 5′-GAAAGCCTTGCAGAGGTCAG-3′, respectively. The sequences for forward and reverse primers for quantifying VIM mRNA were 5′-GCAAAGATTCCACTTTGCGT-3′ and 5′-GAAATTGCAGGAGGAGATGC-3′, respectively. The housekeeping gene Hypoxanthinephophoribosyltransferase-1 (HPRT1) was used as internal reference gene for quantification^41^ as previously described^42,43^. The expression of RNA relative to HPRT1 was calculated^42–44^ based on the Ct as R = 2^−(ΔCt)^, where ΔCt = Ct_target_ − Ct_HPRT1_ mRNA gene expression level was reported as mean ± standard deviation.

### Alizarin Red S staining and quantification of mineralization

The cell monolayers were fixed using 4% formaldehyde after washing gently with PBS. Alizarin red S staining solution at pH 4.1–4.3 was added to the fixed cell monolayers and incubated in the dark for 45 min. Cell monolayers were carefully washed with distilled water and PBS after aspirating the remaining alizarin red S solution. The stained cells were imaged using a camera (Lumenera Infinity 1) mounted on a microscope (Leica DMIL, 0.4 NA and 20 × magnification objective). Several images of the stained cells were captured from three independent cultures for each stable clone, (each frame capturing more than 50 cells) and the pixels corresponding to alizarin red were quantified and normalized to the total number of cells per frame to remove the effects of any differences in cell densities across the clones. The pixels corresponding to the alizarin red stained areas were identified by their RGB decomposition obtained using MATLAB (Mathworks, Natick, MA) and the cells in each frame were counted using the manual mode of Image-J software^45^. The colors determined by the criterion R > 180, R > G + 80 and B < 100 were found to accurately represent the color of the alizarin red S stain.

### *In Vitro* migration assays

Transwell inserts (Costar) with porous polycarbonate membranes with a pore size of 8 μm were used to measure the effect of stable OPN silencing on migration in MDA-MB-231 cells. shRNA knockdown and control clones (1 × 10^5^ cells) suspended in 100 μL of serum-free RPMI were added to the upper chamber of the insert and allowed to migrate across the membranes, which occurred under the influence of RPMI medium with 5% fetal bovine serum as chemoattractant in the lower chamber. After 20 hours, the lower sides of the membranes were fixed in 4% formalin and stained with 0.2% crystal violet solution. After staining, four fields of view for each insert were obtained with an inverted microscope at 10 × magnification. Quantitative measurements of the number of cells migrating across the membrane were obtained by applying an intensity threshold after converting the RBG images to 8-bit grayscale images using Image-J software^45^.

### Experimental animal models: Generation of CTC and LMC

All animal experiments were approved by the Johns Hopkins University Animal Care and Use Program in compliance with the Animal Welfare Act regulations and Public Health Service (PHS) policy. Johns Hopkins University has an approved PHS Assurance and maintains accreditation of our program by the Association for the Assessment and Accreditation of Laboratory Animal Care (AAALAC) International. MDA-MB-231 cells were stably transfected with a construct containing DNA of tdTomato fluorescent protein as previously described^43^. Stably tdTomato-expressing MDA-MB-231 breast cancer cells (2 × 10^6^) were orthotopically implanted into the fourth right mammary fat pad of 6 weeks old female athymic nu/nu female mouse (NCI) as described previously^42,43,46^. When primary tumor volume reached approximately 500 mm^3^ after about 8–12 weeks following inoculation, the mouse was sacrificed, its blood was obtained by cardiac puncture, and its lungs were collected to isolate and culture CTC and LMC, respectively, as briefly described in the following. For CTC isolation, cardiac puncture yielded about 500 μl of blood from each mouse. Red blood cells were lysed (ACK lysing buffer, Life technology, Grand Island, NY, USA) and CTCs were pelleted by centrifugation, suspended in RPMI 1640 culture medium, and CTC presence was verified by fluorescence microscopy of tdTomato-expression in MDA-MB-231 cells. For LMC isolation, both lungs were carefully removed and cut into 4 mm sized tissue pieces with a sterile scalpel and scissors. These lung tissue pieces were placed onto sterile pyrex petri dishes, washed 3 times in balanced salt solution without calcium and magnesium, and finely chopped in 0.25% trypsin/EDTA solution. Lung tissue was digested at 37 °C for 1 hour to maximize trypsin penetration. Warm, complete media was added to the tissue pieces, which was gently dispersed by pipetting. The resulting tissue suspension was then passed twice through a 20 G syringe needle to completely disperse any remaining tissue. Two weeks after cell culture of CTC and LMC, the tdTomato-expressing CTC or LMC cells were sorted by FACS to clear out all non-fluorescent non-cancer cells of lung origin.

### Quantification and statistical analysis

Statistical significance of the differences between quantitative measurements were analyzed by unpaired two-tailed Student’s t-test. P-values < 0.05 were considered to be statistically significant.
